# Evaluation of the Feasibility of Screening Tau Radiotracers Using an Amyloid Biomathematical Screening Methodology

**DOI:** 10.1155/2018/6287913

**Published:** 2018-12-19

**Authors:** Ying-Hwey Nai, Hiroshi Watabe

**Affiliations:** ^1^Division of Radiation Informatics for Medical Imaging, Graduate School of Biomedical Engineering, Tohoku University, Sendai, Japan; ^2^Division of Radiation Protection and Safety Control, Cyclotron and Radioisotope Center, Tohoku University, Sendai, Japan

## Abstract

The purpose of this study is to evaluate the feasibility of extending a previously developed amyloid biomathematical screening methodology to support the screening of tau radiotracers during compound development. 22 tau-related PET radiotracers were investigated. For each radiotracer, in silico MLogP, *V*_x_, and in vitro *K*_D_ were input into the model to predict the in vivo *K*_1_, *k*_2_, and BP_ND_ under healthy control (HC), mild cognitive impaired (MCI), and Alzheimer's disease (AD) conditions. These kinetic parameters were used to simulate the time activity curves (TACs) in the target regions of HC, MCI, and AD and a reference region. Standardized uptake value ratios (SUVR) were determined from the integrated area under the TACs of the target region over the reference region within a default time window of 90–110 min. The predicted *K*_1_, *k*_2_, and BP_ND_ values were compared with the clinically observed values. The TACs and SUVR distributions were also simulated with population variations and noise. Finally, the clinical usefulness index (CUI) ranking was compared with clinical comparison results. The TACs and SUVR distributions differed for tau radiotracers with lower tau selectivity. The CUI values ranged from 0.0 to 16.2, with 6 out of 9 clinically applied tau radiotracers having CUI values higher than the recommend CUI value of 3.0. The differences between the clinically observed TACs and SUVR results showed that the evaluation of the clinical usefulness of tau radiotracer based on single target binding could not fully reflect in vivo tau binding. The screening methodology requires further study to improve the accuracy of screening tau radiotracers. However, the higher CUI rankings of clinically applied tau radiotracers with higher signal-to-noise ratio supported the use of the screening methodology in radiotracer development by allowing comparison of candidate radiotracers with clinically applied radiotracers based on SUVR, with respect to binding to a single target.

## 1. Introduction

Alzheimer's disease (AD) is a progressive neurodegenerative disorder defined by histopathological features such as senile plaques and neurofibrillary tangles (NFT), and clinical symptoms such as memory loss and reduced executive functions [1]. The yearly number of AD cases is increasing worldwide, leading to an increased cost of care for dementia patients. Positron emission tomography (PET) using amyloid and tau radiotracers can measure the amyloid and tau loads, in terms of standardized uptake values ratio (SUVR), and their distributions in a subject's brain from static PET images. Since abnormal accumulation of amyloid and tau in the brain occurs before clinical symptoms appear, the imaging of these precursors can support differential diagnosis and early intervention to increase the success rate of treating AD or slow down the rate of dementia. As such, the 2018 National Institute on Aging-Alzheimer's Association (NIA-AA) research framework includes not only symptomatic stages of AD, but also biomarker classification involving amyloid, tau, and neurodegeneration AT(N) biomarkers [2]. The new framework will be able to identify subjects at risk for AD for suitable and early treatment, in particular, preclinical AD subjects (classified as A+T−(N−) or A+T+(N+)), who are not cognitively impaired but have abnormal amyloid and tau protein deposits [2].

Despite active efforts since 2000 to develop amyloid and tau-targeting PET radiotracers to assist in the diagnosis of AD and to support AD drug development, there are few radiotracers that have made it into clinical studies and displayed good clinical efficacy. In conventional radiotracer and drug development, poor bench-to-bedside translation often results due to the differences between in vitro and in vivo conditions. Similarly, animal models, especially rodents, are often poor predictors of human physiology and treatment response and have been reported to be incorrect in approximately one out of three cases [3]. Although larger animals (e.g., pigs and primates) show closer physiology to that of human, they are still in-prefect human models and are costly for high-throughput screening compared to rodents. These issues lead to high attrition rates in drug and radiotracer development. Biomathematical simulation can complement high-throughput screening by allowing simultaneous and rapid evaluation of many candidate radiotracers [4–6].

Compared to amyloid radiotracers, the development of a successful tau radiotracer encounters additional challenges due to the tau phenotypes. Tau proteins have six isoforms, which differ in the number of exons (0, 1, 2) on the acidic region and the number of repeats (3 repeats (3R) or 4R) in the repeat-domain regions [7]. The different isoforms undergo several posttranslational modifications, leading to various ultrastructural conformations, which will affect the binding of tau radiotracers. In addition, they also need to discriminate between the paired helical filament (PHF) tau from other *β*-sheet structured aggregates such as amyloid-beta (A*β*) and *α*-synuclein. Although the tau protein is larger than the A*β* protein, the tau binding sites are present in smaller concentrations compared to the A*β* binding sites by 5–20 folds; hence, the selectivity of tau over other *β*-sheet structured aggregates needs to be high to ensure accurate quantification. Moreover, as tau proteins exist intracellularly, tau radiotracers not only need to cross the blood-brain barrier (BBB), they also need to be able to cross the cell membrane [8].

Existing clinically applied tau radiotracers showed some limitations. [^11^C]PBB3 has high binding selectivity to tau over A*β*, but it is difficult to synthesize as it will undergo photoisomerization [9]. Moreover, it is rapidly metabolized in the plasma, and its polar metabolite is shown to cross the blood-brain barrier and enter into the brain [10]. The short half-life of carbon-11 has also prompted the development of fluorinated PBB3 compounds ([^18^F]AM-PBB3 and [^18^F]PM-PBB3) and other tau radiotracers so that they can be used in hospitals without dedicated cyclotron facilities. [^18^F]T808 (also known as [^18^F]AV-680) exhibits defluorination, which will affect the quantitative analysis of PET images especially for regions near the skull [11]. Some THK compounds (Tohoku University, Japan) showed differences in the uptake due to the enantiomeric properties of the compounds [12]. A serious confounding factor facing the development of tau radiotracers is off-target brain binding, which might affect the quantitative analysis of the PET images as observed in [^11^C]PBB3, [^18^F]THK5351, and [^18^F]flortaucipir (also known as [^18^F]AV1451 or [^18^F]T807) [13–15]. [^18^F]MK6240 was reported to have reduced off-target binding but further evaluation was still required [16].

We have previously developed an amyloid biomathematical screening methodology to support the screening of candidate amyloid radiotracers during compound development [4, 5]. The screening methodology predicts the standardized uptake values ratios (SUVRs) of different subject conditions of a radiotracer and then compares the clinical usefulness of multiple radiotracers simultaneously in discriminating the subject conditions using a clinical usefulness index (CUI). The CUI was developed to objectively evaluate the clinical usefulness of a radiotracer, based on its binding capability to a single target of interest, in terms of SUVR. The SUVR is a semiquantitative parameter that generalizes the complicated behaviors of tau radiotracers. SUVR is also generally preferred for diagnosis of patients in amyloid and tau imaging; hence, the clinical data are more readily available for comparison. Thus, we chose SUVR over other kinetic parameters such as nondisplaceable binding potential (BP_ND_, unitless).

In this study, we evaluate the feasibility of extending the amyloid-validated screening methodology to support the development of tau PET radiotracers, where more challenges like off-target binding exist. This is the first in silico method investigated, which uses the physicochemical and pharmacological properties of the compounds to support tau PET radiotracers developments. 22 PET radiotracers reported to bind to tau proteins were investigated, including 9 clinically applied and tau-focused radiotracers, namely, [^18^F]THK523, [^18^F]THK5105, [^18^F]THK5117, [^18^F]THK5317, [^18^F]THK5351, [^18^F]flortaucipir, [^18^F]T808, [^11^C]PBB3 and [^18^F]MK6240, and 3 clinically applied but non-tau-focused radiotracers, specifically [^18^F]Lansoprazole, [^11^C]Astemizole, and [^18^F]FDDNP.

## 2. Materials and Methods

An overview of the amyloid biomathematical methodology is described briefly, followed by the screening of tau PET radiotracers using the biomathematical methodology. The details of the methodology are found in somewhere [4, 5].

### 2.1. Biomathematical Screening Methodology

**Figure 1:**
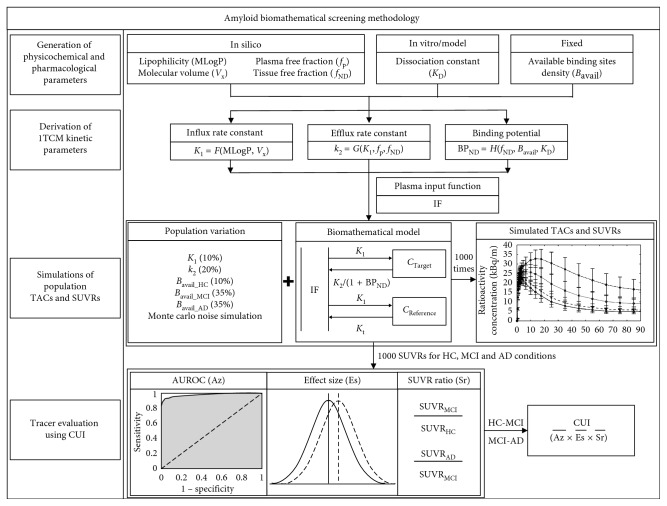
Overview of amyloid biomathematical screening methodology.

The screening methodology was based on a simplified 1-tissue-compartment model (1TCM), with the assumption that the radiotracers cross the blood-brain barrier (BBB) by passive diffusion. It consists of four main parts (Figure 1).

### 2.2. Generation of Physicochemical and Pharmacological Parameters

A total of three inputs were required for each radiotracer: in silico molecular volume and lipophilicity as represented by McGowan Volume (*V*_x_, cm^3^/mol/100), Moriguchi LogP (MLogP, unitless), and an in vitro dissociation constant (*K*_D_, nM) (Table 1). *V*_x_ and MLogP were generated based on the chemical structure of the radiotracer using commercial software, dproperties (Talete, Italy). *K*_D_ values were extracted from the literature, measured via binding assays, using synthetic tau or human brain homogenates. MLogP was used to derive the free fractions of the radiotracer in tissues (*f*_ND_, unitless) and in plasma (*f*_P_, unitless) from the following relationships [4]:

(1) $$\begin{array}{c} f_{\text{ND}}=7.717e^{-1.634\cdot \text{MLogP}},\\ f_{\text{P}}=0.936\cdot f_{\text{ND}}^{0.600}. \end{array}$$

The list of 22 tau radiotracers and their respective inputs are shown in Table 1. The *K*_D_ values that were utilized for simulations are given in bold for human brain homogenates, and italicized for synthetic tau, if available for comparison.

### 2.3. Derivation of 1TCM Kinetic Parameters

The influx rate constant (*K*_1_, mL/cm^3^/min) was derived using the modified Renkin and Crone equation, using compound-specific permeability (*P*, cm/min), with fixed values of capillary surface area (*S* = 150 cm^2^/cm^3^ of brain) and perfusion (*f* = 0.6 mL/cm^3^/min) as follows [4, 6]:

(2) $$\begin{array}{c} K_{1}=f\left(1-e^{-PS/f}\right). \end{array}$$

The compound-specific permeability was derived from the simplified Lanevskij's permeability model, with MLogP and *V*_x_ as inputs [4, 6]:

(3) $$\begin{array}{c} P=\;\;10^{-0.121{\left(\text{MLogP}\;\;-2.298\right)}^{2}\;\;-2.544\log\left(V_{x}^{1/3}\right)-2.525}. \end{array}$$

The efflux rate constant (*k*_2_, min^−1^) can be derived using *K*_1_, *f*_P_, and *f*_ND_ at equilibrium:

(4) $$\begin{array}{c} k_{2}=\;\;\frac{f_{\text{ND}}}{f_{\text{P}}}\cdot K_{1}. \end{array}$$

The in vivo nondisplaceable binding potential (BP_ND_, unitless) was determined using Mintun's equation with *B*_avail_, *f*_ND,_ and *K*_D_:

(5) $$\begin{array}{c} BP_{\text{ND}}=\;\;f_{\text{ND}}\cdot \frac{B_{\text{avail}}}{K_{\text{D}}}. \end{array}$$

The available tau-binding sites (*B*_avail_, nM) were measured using enzyme-linked immunosorbent assay (ELISA). The total amount of tau fibrils (*B*_avail_, nM) in the frontal lobes, parietal lobes, and hippocampus in HC and AD were 1.5 and 16.0 nM, respectively [29], assuming a tau molecular weight of 78,928 Da (https://www.phosphosite.org).

### 2.4. Simulations of Population Time Activity Curves (TACs) and SUVRs

The predicted *K*_1_, *k*_2_, and BP_ND_ were used to simulate the TACs in the target regions of HC, MCI, and AD and a reference region, with a fixed arterial input function (IF):

(6) $$\begin{array}{c} C_{\text{Target}}\left(t\right)=K_{1}\cdot e^{-k_{2}/\left(1+{\text{BP}}_{\text{ND}}\right)\cdot t}\;⊗\text{ IF}\left(t\right),\\ C_{\text{Reference}}\left(t\right)=K_{1}\cdot e^{-k_{2}\cdot t}\;⊗\text{ IF}\left(t\right). \end{array}$$

An input function with similar kinetics to that observed in tau imaging with a fast uptake and washout is required to reflect tau kinetics. For our simulations, a fixed arterial input function was applied with fast kinetics that was derived by averaging the metabolite-corrected arterial plasma input functions of 6 HC subjects injected with [^11^C]BF227 [30].

The same *K*_1_ and *k*_2_ scaling factors of 1.23 and 1.15, respectively, were introduced to account for the differences between the predicted and in vivo values [5]. The scaling factor of BP_ND_ was modified from 0.39 to 1.0 because there were few reported values to determine the appropriate scaling factor. Monte Carlo simulations were applied to generate 1000 TACs in both target and reference regions with 3% noise, to reflect the noise in PET data, and the population variation, by varying *K*_1_ and *k*_2_ by 10% and 20%, respectively [5, 6]. The variations in the tau fibrils in HC and AD were determined as 10% and 35%, respectively, using the ratio of the summed standard deviation to the mean value [29]. The amount of soluble tau in HC, MCI, and AD was reported, but since they did not correlate well with the amount of phosphorylated tau, these values could not be used [31]. In our simulations, the total amount of tau fibrils in MCI was assumed to be the mean of that in HC and AD, with the same amount of variation of 35%, as used for the amyloid simulations [5].

1000 noisy TACs in both target and reference regions were generated by computer simulations with noise. In our simulation, the target region refers to a brain region with varying concentrations of phosphorylated tau depending on subject conditions (e.g., temporal lobe) and a reference is a brain region devoid of phosphorylated tau (e.g., cerebellum). 1000 SUVRs of each subject condition of HC, MCI, and AD were determined from the ratio of the areas under the TACs of the target regions in HC, MCI, and AD and that of the reference region within a chosen time window. For our simulations, a default time window of 90–110 min was selected as the predicted TACs of HC, MCI, and AD appeared to reach a quasi-steady-state in this time window for almost all 9 clinically applied tau radiotracers (Supplementary 2). To evaluate the efficacy of fixed time windows, SUVRs were also determined using the literature-reported time windows for the 9 clinically applied radiotracers.

### 2.5. Tracer Evaluation Using CUI

Az, Es, and Sr are the area under the receiver operating characteristics curve, effect size, and SUVR ratios, respectively. The 1000 SUVR simulated under the subject conditions of HC, MCI, and AD were used to determine Az, Es, and Sr for conditions-pairs of HC-MCI and MCI-AD. CUI was then derived from the product of the averaged Az ($\bar{\text{Az}}$), Es ($\bar{\text{Es}}$), and Sr ($\bar{\text{Sr}}$) of conditions-pairs of HC-MCI and MCI-AD with equal weightage applied:

(7) $$\begin{array}{c} \text{CUI}=\bar{\text{Az}}\times \bar{\text{Es}}\times \bar{\text{Sr}}\text{.} \end{array}$$

The simulated TACs and the predicted SUVR were compared to the clinical data of 9 clinically applied tau radiotracers. The predicted *K*_1_, *k*_2_ and BP_ND_ values were compared with the clinically observed values where applicable. Finally, the list of 22 tau radiotracers (Table 1) was evaluated using CUI. We previously developed a MATLAB-based program, RSwCUI, (Ver. 2014b, The MathWorks, US) [5], to support the screening of amyloid radiotracers based on the proposed amyloid biomathematical screening methodology. The program was used for the evaluation of tau radiotracers in this study.

## 3. Results

**Figure 2:**
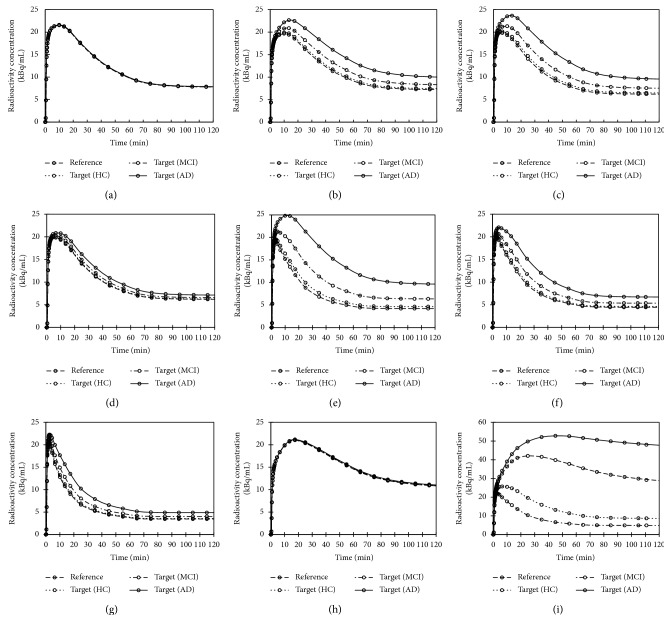
Simulated TACs of target regions of HC, MCI, and AD and reference regions for (a) [^18^F]THK523, (b) [^18^F]THK5105, (c) [^18^F]THK5117, (d) [^18^F]THK5317, (e) [^18^F]THK5351, (f) [^11^C]PBB3, (g) [^18^F]flortaucipir, (h) [^18^F]T808, and (i) [^18^F]MK6240 from 0–120 min.

Figure 2 shows the simulated TACs for the target regions of HC, MCI, and AD and reference regions of 9 clinically applied tau radiotracers. In general, the clinically observed TACs of THK compounds of the reference region had higher peaks and faster washout in the cerebellum than the target regions [15,32–35], while the peaks of the simulated TACs of the reference region were always lower than that of the target regions (Figures 2(a)–2(e)). The simulated TACs of [^11^C]PBB3 (Figure 2(f)) were close to that observed clinically in AD in the nonbinding and low-, middle-, and high-binding regions [10]. The simulated TACs of [^18^F]flortaucipir (Figure 2(g)) had slightly sharper peaks and faster washout compared to the clinically observed TACs for both HC and AD [36]. Unlike the THK compounds, the peaks of the clinically observed TACs of the target regions of [^18^F]flortaucipir were higher than that of the reference region, which was also observed in the simulated TACs [36]. The predicted TACs of [^18^F]T808 for both the reference and the target regions of HC, MCI, and AD conditions completely overlapped with each other (Figure 2(h)). The clinically observed TACs of [^18^F]T808 appeared close to that of [^18^F]flortaucipir, but with smaller differences between the subject conditions. However, the simulated TACs showed complete overlapped between the HC and AD conditions with a slower uptake and washout [37]. The predicted TACs of both target and reference regions of [^18^F]MK6240 showed similar fast uptake but slower washout than clinically observed TACs [16].

Table 2 compares the predicted and clinically-reported values of *K*_1_, *k*_2_, and BP_ND_ of five clinically applied tau radiotracers with reported kinetic parameters. For [^18^F]flortaucipir, the predicted *K*_1_ and *k*_2_ values of 0.256 and 0.199, respectively, were relatively close to the reported averaged cerebellar *K*_1_ and *k*_2_ values of 0.26 and 0.17, respectively [36]. The predicted *k*_2_ value of [^18^F]THK5351 was 0.140, which was higher than the clinically observed value of 0.115, with a difference of 21.7% [38]. However, unlike [^18^F]flortaucipir where both *K*_1_ and *k*_2_ values were determined using the two-tissue-compartment model with a variable fraction [36], the reported *k*_2_ value of [^18^F]THK5351 was an apparent rate constant from reference region to plasma, which was determined using the simplified reference tissue model (SRTM) [38].

The predicted *k*_2_ value of [^18^F]THK5317 of 0.087 was close to the literature-reported value of 0.09, even though *K*_1_ of [^18^F]THK5317 value differed of [^18^F]THK5317 from the clinically observed value with a difference of −39% [39]. The predicted BP_ND_ values of 0.125 and 8.13 were very different from the clinically observed values of 0.60 and 5.11 in AD for [^18^F]THK5317 [39] and [^18^F]MK6240 [16]. The predicted BP_ND_ value was fairly close to that of [^11^C]PBB3 [10]. The predicted *K*_1_ of [^18^F]MK6240 was close to the clinically observed *K*_1_ value with 2.50% difference but the predicted *k*_2_ value yielded greater difference of about 40% [16].

Table 3 shows the predicted SUVR values obtained using the default time window and literature-reported time window of 90–110 min, and the clinically observed SUVR for 10, 10, and 9 clinically applied tau radiotracers. The differences in the SUVRs predicted using both time windows were very small for both HC and AD. The predicted SUVR for HC was always greater than 1.0, but the clinically observed SUVR values were less than 1.0 for some radiotracers. In general, the clinically observed SUVR for HC and AD were greater than the predicted SUVR determined using the literature-reported time window, except for [^11^C]PBB3 and [^18^F]MK6240, where the predicted SUVR for HC and AD were greater.

**Figure 3:**
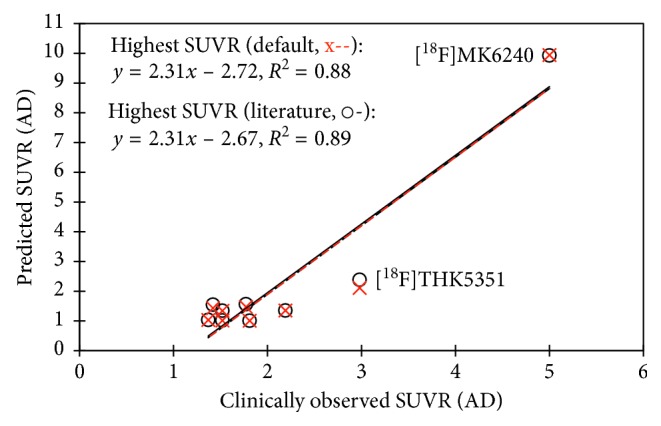
Correlations of clinically observed and predicted SUVR values using literature-stated time window (o-) and default time window (x--).

The correlations between the predicted and highest clinically observed SUVR for AD were similar with coefficients of determination, *R* ^2^ of 0.90 and 0.89, respectively, using the literature-reported time window and the default time window (Figure 3). However, the good correlation was driven by [^18^F]MK6240, which had the highest predicted and clinically observed SUVR. Poor correlation was observed after removing [^18^F]THK5351 and [^18^F]MK6240. The small difference between the predicted SUVR using the default and clinical-reported time window, and the value of *R* ^2^, showed that the default time window of 90–110 min was suitable for predicting the SUVR of the tau radiotracers (Figure 3).

**Figure 4:**
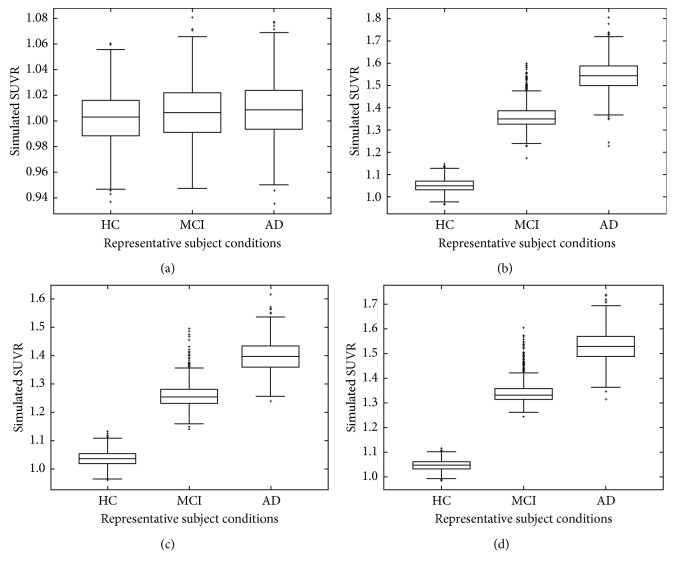
Simulated SUVR distributions of (a) [^18^F]THK523, (b) [^18^F]THK5117, (c) [^18^F]flortaucipir, (d) [^11^C]PBB3.

The simulated SUVR distribution of [^18^F]THK523 across HC, MCI, and AD conditions substantially overlapped each other (Figure 4(a)). However, the clinically observed SUVR distribution of [^18^F]THK523 differed across different regions of interest, with HC− (PIB-negative) having the smallest spread and smallest values, HC+ (PIB-positive) having a relatively large spread and values ranging between that of HC− and AD, and AD subjects having the largest values and a nearly similar spread as HC+ [30]. For [^11^C]PBB3, [^18^F]THK5117, and [^18^F]flortaucipir, the clinically observed SUVR distributions were generally larger for AD than HC for all regions of interest analyzed, in terms of the spread and absolute values [14, 34, 40]. The trend of the simulated SUVR population distribution was close to that observed clinically for HC and AD conditions (Figures 4(b)–4(d)). This supported the use of 35%, 35% and 10% variations in *B*_avail_ for population simulations.

**Figure 5:**
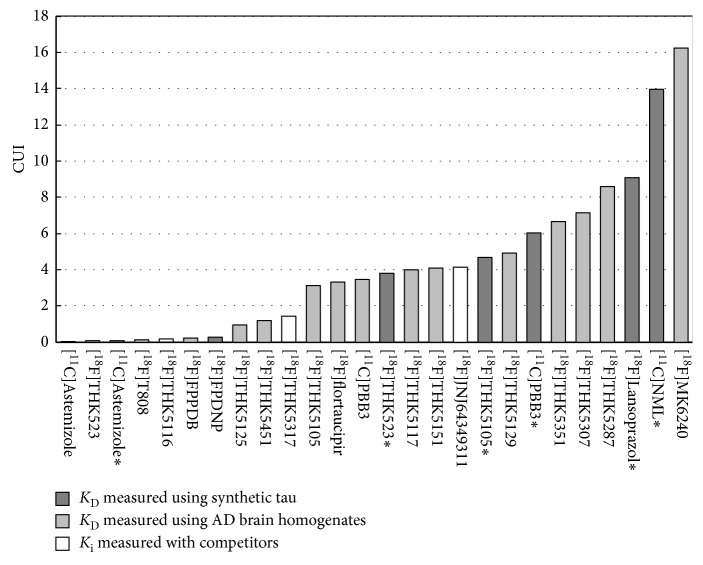
CUI distributions of 22 tau-related PET radiotracers.

Figure 5 shows the CUI distribution of 22 tau-related radiotracers. Among the clinically applied tau radiotracers, [^18^F]MK6240 was ranked first, followed by [^18^F]THK5351, [^18^F]THK5117, [^11^C]PBB3, [^18^F]flortaucipir, [^18^F]THK5317, [^18^F]FDDNP, [^18^F]T808, and [^18^F]THK523, based on the *K*_D_ values measured using AD brain homogenates. For candidate radiotracers, [^18^F]THK5287 was ranked first based on the *K*_D_ values measured using AD brain homogenates, while [^11^C]NML was ranked first based on the *K*_D_ values measured using heparin-induced tau polymer (HITP) (Table 1). The CUI values generated using the *K*_D_ values for the synthetic tau were higher than those of the brain homogenates as the *K*_D_ values measured using synthetic tau were smaller (Table 1). The ranking of the CUI values generated using the *K*_D_ values measured with synthetic tau and brain homogenates differed for [^18^F]THK523, [^18^F]THK5105, and [^11^C]PBB3. 10 out of 16 tau radiotracers had CUI values higher than the recommend CUI value of 3.0, where the results were simulated using *K*_D_ values measured with human brain homogenates. Apart from [^18^F]THK523, [^18^F]THK5317, [^18^F]T808, and [^18^F]FDDNP, the other 6 clinically applied tau radiotracers yielded high CUI values. The CUI values ranged from about 0.0 to 16.2, which ranged wider than that for amyloid.

## 4. Discussion

In this paper, we evaluated the feasibility of extending a previously developed amyloid biomathematical screening methodology to support the screening of candidate tau radiotracers during compound development. 22 clinically applied and candidate tau-related radiotracers were thus used to investigate the CUI ranking of clinically applied and candidate tau radiotracers.

### 4.1. Comparison of Simulated TACs and SUVR Distribution

The simulated TACs were very different from the clinically observed TACs of [^18^F]THK523 and [^18^F]T808, but were only slightly different for that of [^18^F]THK5117, [^18^F]THK5351, [^18^F]flortaucipir, [^11^C]PBB3, and [^18^F]MK6240 (Figure 2). The simulated SUVR distributions were different for [^18^F]THK523 but were similar to the clinically observed results under HC and AD conditions for [^18^F]THK5117, [^18^F]flortaucipir, and [^11^C]PBB3 (Figure 4). Both the predicted and clinically observed SUVR values were less than 1.0 in HC for some radioligands, especially those with a lower selectivity for tau (e.g., [^18^F]THK523). The clinically observed SUVR of AD is much higher than that of HC. However, there is little difference in the predicted SUVR for [^18^F]THK523. This shows that the predictions were less accurate for tau compounds with a lower selectivity for the target. Poor predictions might be due to binding to other *β*-sheet structured proteins or off-target sites shown in the clinical data, whereas the predicted values showed the binding of the radiotracers to only the target site. Nonspecific binding in white matter may also lead to spill-over into the surrounding cortical regions, leading to higher clinically observed SUVRs. The issue of non-specific binding is more apparent for tau radiotracers with lower tau-binding selectivity, such as [^18^F]THK523 and [^18^F]THK5117 (Table 2).

### 4.2. Comparison of Predicted 1TCM and SUVR

The prediction for the *K*_1_ and *k*_2_ values of the tau radiotracers appeared to work well in racemic compounds (e.g., [^18^F]flortaucipir), but not as well for enantiomeric compounds like [^18^F]THK5351 and [^18^F]THK5317, which are S-enantiomers of [^18^F]THK5151 and [^18^F]THK5117 respectively (Table 2). The predictions for BP_ND_ were generally poor for the three clinically-reported tau radiotracers (Table 2). This may be due to the use of a simplified 1TCM for prediction, even though 2TCM was reported to be more suitable for many clinically applied tau radiotracers. The simplified 1TCM was selected even though 2TCM is more accurate for modeling tau kinetics as the prediction of a larger number of microparameters may be difficult to estimate reliably. Moreover, the 1TCM worked reasonably well in predicting the kinetics of the amyloid radiotracers, even though 2TCM was reported to be more suitable [5]. Other possible reasons for the poorer BP_ND_ predictions included differences in binding to the plasma proteins due to the enantiomeric properties of the radiotracers [42], metabolites crossing the BBB for [^11^C]PBB3 [10], binding of tau radiotracers to other similar *β*-sheet structures (A*β* and α-synuclein), or off-target binding in target regions of interest [13–15]. The predicted 1TCM parameters and SUVR, as well as the simulated TACs and SUVR distribution, were compared to clinically observed data where applicable. However, we were limited by the small number of reported kinetic parameters and SUVR values to fully assess the amyloid biomathematical model for screening tau radiotracers.

The predicted and highest clinicallyobserved SUVR data for AD correlated well using fixed time window of 90–110 min and the literature-reported time window with *R* ^2^ values of 0.88 and 0.89 respectively, for 9 clinically applied tau radiotracers (Figure 3). However, the results were driven mostly by [^18^F]MK6240. Some of the clinically applied tau radiotracers ([^18^F]THK523, [^18^F]THK5351 and [^18^F]flortaucipir) did not have high selectivity for tau, which may have contributed to smaller predicted values as the predicted values were based on binding to a single target site but the off-target binding or specific binding to other *β*-sheet structures (e.g., amyloid) may yield higher clinical SUVR values. The predicted TACs of [^18^F]T808 exhibited a much slower clearance compared to the clinically observed kinetics, which resulted in a large difference between the predicted and clinically observed SUVR. This may be due to the poor predictive ability of in silico parameters for [^18^F]T808, which has a unique chemical structure.

### 4.3. Comparison of Tau Radiotracers with CUI

The CUI value of [^18^F]flortaucipir was large while the CUI value of [^18^F]T808 was very small and does not appear to be a promising clinical tau radiotracer. Similarly, [^18^F]THK523 also yielded a small CUI value, even though studies showed that it could be applied clinically. [^18^F]THK523, [^18^F]Lansoprazole, and [^11^C]Astemizole yielded small CUI values using the *K*_D_ values measured using human brain homogenates, which differed greatly from that measured using synthetic tau. *K*_D_ or *K*_i_ values measured using AD brain homogenates were very different from those measured using heparin-induced tau polymer (HITP) (Table 1). This is because HITP is composed of only 3R and/or 4R, and hence may not undergo the same phosphorylation process as human tau [19, 43]. On the other hand, the *K*_D_ or *K*_i_ values of amyloid radiotracers measured using synthetic tau and AD brain homogenates did not differ greatly [5]. The huge difference in the *K*_D_ values measured using human brain homogenates and synthetic tau were much greater for [^18^F]THK523 than for [^18^F]THK5105 (Table 1). This might also indicate the binding preferences of [^18^F]THK523 to certain tau-binding sites available on synthetic tau, that were fewer in numbers in human brain homogenates. Therefore, it is important to determine the binding affinity of tau radiotracers to different subtypes of tau protein and other *β*-sheet structures such as A*β* and *α*-synuclein.

[^18^F]THK5351 yielded higher clinically observed SUVR than [^18^F]THK5117 in the same AD patients, with lower white matter binding [15]. [^18^F]THK5351 was also reported to have a higher signal-to-noise ratio (SNR), and a lower non-specific binding in white matter than [^18^F]THK5105 and [^18^F]THK5117 [8]. Similarly, the CUI value of [^18^F]THK5351 was higher than [^18^F]THK5105 and [^18^F]THK5117. [^11^C]PBB3, [^18^F]flortaucipir, and [^18^F]THK5105 have nearly similar CUI values (Figure 5), but the difference in the clinically observed SUVR values between HC and AD were greatest in [^18^F]flortaucipir, followed by [^11^C]PBB3 then [^18^F]THK5105 (Table 3). This difference may be attributed to the tau subtypes that [^11^C]PBB3 is binding. [^18^F]THK5351 and [^18^F]flortaucipir was reported to bind to the same targets but with different affinities, while [^11^C]PBB3 seems to bind to a different tau subtype [44]. If the tau subtype that [^11^C]PBB3 binds to is of a lower concentration in subject, the clinical SUVR will become smaller. The difference between the clinically observed results and CUI ranking showed that the evaluation of the clinical usefulness of tau radiotracer based on binding to a single target could not reflect the actual in vivo binding in subjects. High tau selectivity and off-target binding affect the comparison of the in vivo binding of tau radiotracers, which are less prominent in amyloid radiotracers. Despite the differences in CUI rankings, the clinically applied tau radiotracers had CUI values above the recommended value especially for those with high SNR. Thus, the screening methodology can still provide confidence in the decision-making of moving candidate radiotracers for clinical studies.

### 4.4. Limitations of Screening Methodology

Few measurements of tau concentration in postmortem human brains using ELISA have been reported, and these values are very different [17, 29, 45, 46]. In addition, these reported tau concentrations were mostly measured using normal-aged control and AD brains, with very little data on the tau concentration in MCI. As such, the simulated SUVR distribution might not reflect the clinically observed MCI result. Moreover, the input function of the amyloid radiotracer [^11^C]BF227 was used for simulations. Thus far, the input functions of only three clinically applied tau radiotracers of [^11^C]PBB3 [10], [^18^F]flortaucipir [36], and [^18^F]MK6240 [16] have been reported. The arterial input functions of these radiotracers were similar in HC and AD, with a fast uptake and a fast washout, and the shape of the curves was similar to that of [^11^C]BF227 as used in the simulation. Although the shape of the input function of these two radiotracers was similar to that of [^11^C]BF227, the shape of the arterial input function might be different for other tau radiotracers. Thus, we evaluated the effect of the input function on the outcome using four different input functions with fast kinetics for HC and AD subjects injected with [^11^C]BF227 or [^18^F]FACT, with areas under the input function curves from 0 to 120 min of 536 (default), 649, 434, and 306 (kBq/mL) min. The %COV of the predicted SUVR was less than 7.0 for all conditions and radiotracers, while %COV of the CUI was less than 7.0 for all except the poor radiotracers, namely, [^18^F]FDDNP, [^18^F]FPPDB, and [^11^C]Astemizole. This showed that the results would not be changed significantly using input functions with similar kinetics. However, there were also issues with metabolites crossing the BBB (e.g., [^11^C]PBB3), but the amyloid biomathematical screening methodology could not be used to predict the possibility of metabolites crossing this barrier.

Off-target binding was observed in some clinically applied tau radiotracers. [^18^F]flortaucipir was reported to show specific binding in the midbrain, vessels, iron-associated regions (e.g., basal ganglia), substantia nigra, calcifications in the choroid plexus, and leptomeningeal melanin [13]. [^11^C]PBB3 was reported to accumulate in the venous sinuses, basal ganglia, and thalamus, while its fluorinated compounds showed off-target binding in the choroid plexus [14, 44]. [^18^F]THK5351 was reported to bind to monoamine oxidase B (MAO-B), which is highly expressed throughout the brain, and thus, its tau binding data needs to be corrected for MAO-B binding [47]. [^18^F]MK6240 was reported to have reduced off-target binding on the whole but showed off-target binging in regions such as the retina, substantia nigra, ethmoid sinus, and dura matter [16]. Depending on the region of off-target binding, the effects may not limit PET quantification due to little or no anatomical overlap of the target regions of interest (ROIs) with off-target regions. Accurate PET quantification is also less affected if the radiotracer has high target selectivity or if the concentrations of the off-target binding sites are much lower compared to that of the target [48]. Off-target binding may be one of the contributing factors that led to the observed differences between simulation and the clinical data of tau PET radiotracers. The possibility of binding to off-targets is difficult to predict, and systematic screening is required to determine the binding of the candidate compound to a wide range of proteins. This will increase the time and cost of compound screening. The amyloid biomathematical screening methodology could not predict off-target binding, and the inclusion of multiple binding sites appeared to be required for tau radiotracers to correct for this issue.

### 4.5. Feasibility of Extending to the Screening of Tau Radiotracers

To date, the comparison of multiple tau radiotracers has been performed via in vitro competition binding assays in human brain sections, using human AD brain homogenates [11, 12] or by means of preclinical imaging [38]. There is a lack of consideration of the possible in vivo kinetics of the radiotracers during development, which may lead to poor clinical performance [4–6]. The use of in silico data can support predictions of tracer kinetics and increases confidence in clinical translation, in addition to facilitating radiotracer comparisons. The weak SUVR correlation was obtained between the predicted and clinically observed SUVR results, mostly due to the small SUVR values for tau radiotracers with poorer tau selectivity. However, there are very few reported kinetic parameters to assess the limitations of the screening methodology. The TACs, SUVR distribution, and CUI rankings differed primarily for tau radiotracers with low selectivity to tau. This showed that the evaluation of the clinical usefulness of tau radiotracer based on binding to a single target could not fully reflect the actual in vivo binding in subjects since they also exhibited binding preferences to nontarget sites. Thus, it is not feasible to directly apply the amyloid biomathematical screening methodology to tau radiotracers due to the increased complexity of evaluating the binding of tau radiotracers, namely, target-binding, off-target binding, and non-specific binding. More work is required to improve the accuracy of predicting the clinical usefulness of tau radiotracers by including possible binding to other *β*-sheet structures or off-target sites. However, the high CUI values generated for clinically applied tau radiotracers with high SNR showed that the screening methodology could be used to increase confidence in decision-making when choosing candidate radiotracers for further evaluation.

## 5. Conclusions

The predicted TACs, SUVR, and CUI ranking differed for some clinically applied tau radiotracers, especially those with lower selectivity for tau. This showed that the evaluation of the clinical usefulness of tau radiotracer based on binding to a single target could not reflect the actual in vivo tau binding in subjects due to more challenges in evaluating the in vivo binding of tau radiotracers, such as off-target binding and high tau selectivity, compared to amyloid radiotracers. The inclusion of possible binding to other *β*-sheet structures or off-target sites and the binding affinities to different target sites would improve the accuracy of the prediction. From our results, clinically applied tau radiotracers with higher SNR, such as [^18^F]MK6240 and [^18^F]THK5351, had higher CUI rankings. This supported the use of the screening methodology in radiotracer development by allowing comparison of candidate radiotracers with clinically-applied radiotracers based on SUVR, with respect to binding to a single target. Our results will hopefully provide some insights to guide the development of in silico models in supporting the development of tau radiotracers.

## Figures and Tables

**Table 1:** In silico MLogP and *V*_x_ and in vitro *K*_D_ of 22 tau-related PET radiotracers. *K*_D_ values employed for simulations are given in bold (measured using brain homogenates) and italicized (measured using synthetic tau).

Radiotracers	MLogP	*V*_x_	*K*_D_	References for *K*_D_
[^18^F]THK523	3.19	2.11	*1.67* ^*α*^	[17]
			1.99^*α*^	[18]
			**86.5**	[19]
[^18^F]THK5105	3.08	2.59	*1.45* ^*α*^	[19]
			**2.63**	
[^18^F]THK5116	2.62	2.31	**106^&^**	[12]
[^18^F]THK5117	2.85	2.45	**2.65^$^**	[20]
[^18^F]THK5125	3.08	2.59	**10.2**	[12]
[^18^F]THK5129	2.48	2.55	**3.14**	[12]
[^18^F]THK5151	2.25	2.41	**7.07**	[12]
[^18^F]THK5287	1.94	2.55	**2.60**	[12]
[^18^F]THK5307	1.71	2.41	**5.60**	[12]
[^18^F]THK5317	2.85	2.45	**9.40^&^**	[21]
[^18^F]THK5351	2.25	2.41	**2.90**	[15]
[^18^F]THK5451	2.25	2.41	**28.0**	[12]
[^18^F]flortaucipir	1.95	1.86	**14.6^#^**	[22]
[^18^F]T808	3.64	2.23	**22.0** ^#^	[11]
[^11^C]PBB3	2.34	2.31	*2.50* ^*α*^	[10]
			**6.30**	[23]
[^18^F]FDDNP	2.89	2.31	*36.7* ^*α*^	[18]
[^18^F]FPPDB	2.87	3.15	**44.8**	[24]
[^11^C]NML	1.98	2.51	*0.700* ^*α*^	[25]
[^18^F]Lansoprazol	1.75	2.37	*3.30* ^*α*^	[25]
			**>3998^*δ*^**	[11]
[^11^C]Astemizole	4.63	3.56	**13.4**	[26]
			*1.86^α^*	
			>3998^*δ*^	[11]
[^18^F]MK6240	2.49	1.96	**0.260^*β*^**	[27]
[^18^F]JNJ64349311 ([^18^F]JNJ311)	2.07	1.83	**7.90^*δ*^**	[28]

Units: MLogP (unitless), *V*_x_ (cm^3^/mol/100), *K*_D_ (nM). ^$^Averaged *K*_D_ values (2.2, 3.1) for tau in AD brain homogenates of temporal and hippocampus. ^*β*^Averaged *K*_D_ values 0.14, 0.30, 0.25, 0.24, and 0.38 for tau in AD brain homogenates of frontal and entorhinal cortex of 5 AD. ^*α*^ *K*_D_ values are measured using synthetic tau (K18Δ280K) ^&^ *K*_i_ values measured using AD brain homogenates with THK5105 as competitor ^*δ*^ *K*_i_ values measured using AD brain homogenates with T808 as competitor. ^#^ *K*_D_ values measured using AD brain via autoradiography.

**Table 2:** Comparison of predicted and clinically observed *K*_1_, *k*_2_, and BP_ND_ values of four clinically applied tau radiotracers.

Radiotracers	Literature				Predicted values	% diff
	Parameters	Region	Clinically observed values	References		
[^18^F]flortaucipir	*K*_1_	Cerebellum excluding vermis	0.26	[36]	0.256	−1.54
	*k*_2_		0.17		0.199	17.1
[^18^F]THK5351 (*S*-enantiomer of [^18^F]THK5151)	*k*_2_′^‡^	Target^*β*^	0.115	[38]	0.140	21.7
[^18^F]THK5317 (*S*-enantiomer of [^18^F]THK5117)	*K*_1_	Target^*δ*^	0.33	[35]	0.202	−38.8
	*k*_2_		0.09		0.087	−3.33
	BP_ND_ (AD)^*∗*^	Putamen	0.60	[39]	0.125	−79.2
[^11^C]PBB3	BP_ND_ (AD)^¶^	High-binding cortical regions	0.37	[10]	0.427	15.4
[^18^F]MK6240	*K*_1_	Posterior cingulate cortex	0.246	[16]	0.252	2.50
	*k*_2_		0.099		0.138	39.2
	BP_ND_^§^		5.11		8.13	59.2

^*β*^Target ROIs: anterior cingulate, brainstem, caudate nucleus, eroded white matter, entorhinal cortex, frontal cortex, fusiform gyrus, hippocampus, inferior temporal cortex, lingual gyrus, middle temporal gyrus, occipital cortex, pallidum, parahippocampal gyrus, parietal cortex, posterior cingulate, precuneus, putamen, thalamus. ^*δ*^Target ROIs: thalamus, putamen, hippocampus, amygdala, parietal cortex, frontal cortex, sensory motor cortex, occipital cortex, midbrain, entorhinal cortex, and temporal cortex. ^*∗*^BP_ND_ = DVR-1, where DVR was determined using reference Logan, averaged from 4 prodromal AD. ^¶^BP_ND_ determined using MRTM_0_. ^§^BP_ND_ determined using *k*_3_/*k*_4_ using 2T4CM in 7 symptomatic individuals classified as MCI and AD. ^‡^ *k*_2_′ optimized from fitting all target ROIs using SRTM with cerebellum as the reference region.

**Table 3:** Comparison of predicted (literature-reported and default time window of 90–110 min) and clinically observed SUVR (highest SUVR in AD) of HC and AD conditions.

Clinically applied radiotracers	Predicted SUVR				Clinically observed SUVR				
	Default		Literature		Highest in AD		Regions	Time window (min)	References
	HC	AD	HC	AD	HC	AD			
[^18^F]THK523	1.00	1.01	1.00	1.01	0.96	1.81	ITL	60–90	[32]
[^18^F]THK5105	1.03	1.34	1.03	1.35	1.41	1.52	PU	90–100	[33]
[^18^F]THK5117	1.04	1.47	1.05	1.56	1.57	1.77	PU	50–60	[34]
[^18^F]THK5317	1.01	1.13	1.01	1.14	—	—	—	—	—
[^18^F]THK5351	1.10	2.11	1.11	2.38	2.14	2.98	HIP	50–60	[15]
[^18^F]flortaucipir	1.03	1.35	1.03	1.35	1.17	2.19	ITL	80–100	[40]
[^18^F]T808	1.00	1.02	1.00	1.02	0.94	1.52	LTL	80–100	[35]
[^11^C]PBB3	1.04	1.43	1.05	1.55	0.85	1.42	Global^#^	30–50	[10]
[^18^F]FDDNP	1.00	1.03	1.00	1.04	1.24	1.37	ACG	45–55	[41]
[^18^F]MK6240	1.78	9.94	1.78	9.93	—	∼5^*∗*^	PRE	90–110	[16]

ITL = inferior temporal lobe, LTL = lateral temporal lobe, PU = putamen, PAR = parietal lobe, HIP = hippocampus, ACG = anterior posterior cingulate, PRE = precuneus. ^#^Global = cerebral cortex for HC and high binding ROI for AD. ^*∗*^SUVR is approximated from the plot, taking the highest SUVR in AD.

## Data Availability

The program (RSwCUI) used for TACs simulation and CUI evaluation can be download from http://www.rim.cyric.tohoku.ac.jp/software/CUI-Software. The predicted *K*1, *k*2, and BP_ND_ values in HC and AD of 9 clinically applied tau-related radiotracers are provided in the interest of readers and are included within the supplementary information file.

## Supplementary Materials

Supplementary 1 Table 1: Predicted *K*1, *k*2, and BP_ND_ values in HC and AD of 9 clinically applied tau-related radiotracers.

Supplementary 2 Figure 1: Quasi-steady-state of TACs of HC (gray), MCI (blue), and AD (orange) conditions of 9 clinically applied tau radiotracers from 0 to 120 min. The box highlighted the time window of 90 to 110 min, which was applied as the default time window for simulating SUVRs.
