# Supplementary material for: Evaluation of the Feasibility of Screening Tau Radiotracers Using an Amyloid Biomathematical Screening Methodology
Source: Comput Math Methods Med. 2018 Dec 19;2018:6287913. doi: 10.1155/2018/6287913 (PMC6314003; doi:10.1155/2018/6287913)
Supplement: Supplementary 1 — Table 1: Predicted K1, k2, and BPND values in HC and AD of 9 clinically applied tau-related radiotracers. [file 6287913.f1.pdf]

Supplementary Table 1: Predicted K_1, k_2 and BP_{ND} values in HC and AD of 9 clinically-applied tau-related radiotracers.

Clinically-applied Tau Radiotracers	K_1 (mL/cm^3/ min)	k_2 (min^{-1})	BP_{ND} (Unitless)	
			HC	AD
[^{18}F]THK523	0.200	0.069	0.035	0.43
[^{18}F]THK5105	0.181	0.067	0.053	0.67
[^{18}F]THK5117	0.202	0.087	0.042	0.53
[^{18}F]THK5317	0.202	0.087	0.012	0.15
[^{18}F]THK5351	0.220	0.141	0.10	1.28
[^{18}F]flortaucipir	0.256	0.199	0.033	0.42
[^{18}F]T808	0.151	0.039	0.0010	0.0017
[^{11}C]PBB3	0.226	0.136	0.041	0.51
[^{18}F]FDDNP	0.209	0.088	0.003	0.036
